# Struma ovarii: A case report and review the literature

**DOI:** 10.1016/j.ijscr.2022.107318

**Published:** 2022-06-18

**Authors:** Fatemeh Zamani, Aghdas Abdolrazaghnejad, Fereshteh Ameli, Sanaz GHashghaee, Saina Nassiri, Narges Zamani

**Affiliations:** a Department of Radiology, Children Medical Centre of Excellence, Tehran University of Medical Science, Tehran, Iran; b Obstetrics and Gynecology Department, Pregnancy Health Research Center, Ali IbneAbitaleb Hospital, Faculty of Medicine, Zahedan University of Medical Sciences, Zahedan, Iran; c Department of Pathology, Cancer Institute, Imam Khomeini Hospital Complex, Tehran University of Medical Science, Iran; d Department of Obstetrics & Gynecology, Vali-e-Asr Hospital, Tehran University of Medical Sciences, Tehran, Iran.; e Department of Gynecology and Obstetrics, Faculty of Medicine, Tehran University of Medical Science, Tehran, Iran; f Department of Oncologic Gynecology, Vali-e-Asr Hospital, Tehran University of Medical Science, Tehran, Iran

**Keywords:** ORADS, Ovarian-Adnexal Reporting and Data System, CA125, Cancer Antigen 125, ROMA, Risk of Ovarian Malignancy Algorithm, HE4, Human Epididymis Protein 4, CA19-9, Cancer Antigen 19-9, BHCG, Beta Human chorionic gonadotropin, CEA, Carcinoembryonic Antigen, AFP, Alpha Fetoprotein, IHC, Immunohistochemistry, CK7, Cytokeratin 7, TTF1, Transcription Termination Factor 1, PAX8, Paired Box 8, Struma ovarii, Ovarian tumor, Case report, Teratoma

## Abstract

**Introduction and importance:**

Struma ovarii is a monodermal teratoma which characterized by the presence of thyroid tissue. The symptoms of this tumor are nonspecific and thus misdiagnosis and indifference to other ovarian lesions are very common.

**Case presentation:**

Herein, we described a case of struma ovarii that was successfully diagnosed and managed. The tumor is mimicking a malignant tumor based on ascites and tumor marker assessments. Although, thyroid function indices are normal.

**Clinical discussion:**

The initial footprint of the tumor is mostly based on incidental imaging, but definitive diagnosis is possible based on pathological studies. Surgical resection of the tumor can be led to successful treatment and prognosis.

**Conclusion:**

Struma ovarii is a rare tumor and also misdiagnosis is common. Regarding rarity of Struma Ovarii, the treatment option is debated. However, in postmenopausal cases with the aim of completely removing the symptoms, total abdominal hysterectomy with bilateral salpingo-oophorectomy can be occasionally indicative.

## Highlights

• Struma Ovarii is a monodermal ovarian teratoma.
• The symptoms of Struma Ovarii are nonspecific.
• Struma ovarii is a misdiagnosis with other ovarian lesions commonly.
• Surgical resection of Struma Ovarii can be led to successful treatment and prognosis.

## 1 Introduction

Struma ovarii is a rare ovarian lesion which characterized by the presence of thyroid tissue in at least half of the overall ovarian mass [1]. This tumor is considered a teratoma, but sometimes may be encountered with mucinous or serous cystadenomas [2]. This mass comprises less than 1 % of ovarian tumors and also 2 to 5 % of all ovarian teratomas [3]. The symptoms are nonspecific and thus misdiagnosis to other ovarian lesions is very common [4]. The initial footprint of the tumor is mostly based on incidental imaging, but definitive diagnosis is possible based on pathological studies. Surgical resection of the tumor followed by adjuvant radioiodine therapy has been accepted as the definitive therapeutic approach leading prevention of disease metastasis or recurrence [5]. Herein, we described a case of struma ovarii that was successfully diagnosed and managed. Written informed consent was obtained from the patient for publication of this case report and accompanying images. A copy of the written consent is available for review by the Editor-in-Chief of this journal on request.

This work has been reported in line with the SCARE 2020 criteria [6].

## 2 Case presentation

**Fig. 1:**
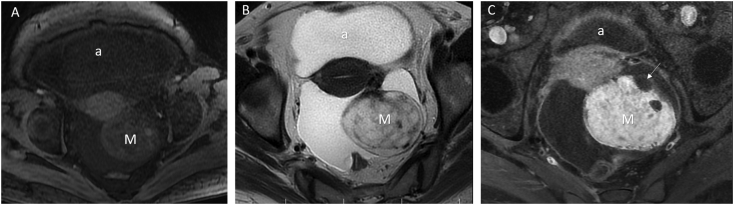
(A) Axial T1-weighted showed a well-defined heterogeneous hypo-to isointense solid mass in left ovary. (B) In axial T2-weighted mass appeared hyper intense and contains multiple thick septa. (C) Post contrast T1-weighted fat-suppression manifested avid enhancement in solid component. a = ascites, M = mass, Arrow = follicule.

**Fig. 2:**
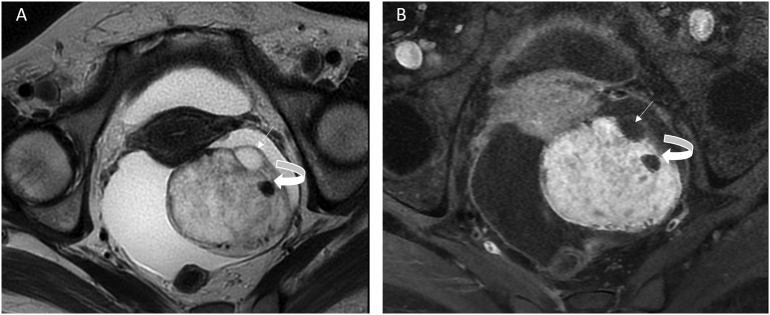
(A&B) T2-weighted and post contrast fat-sat T1-weighted showed a very low signal intensity (curved arrow), due to the viscous colloid material that could be suggestive for the presence of struma ovarii tumor. Thin arrow = follicule.

**Fig. 3:**
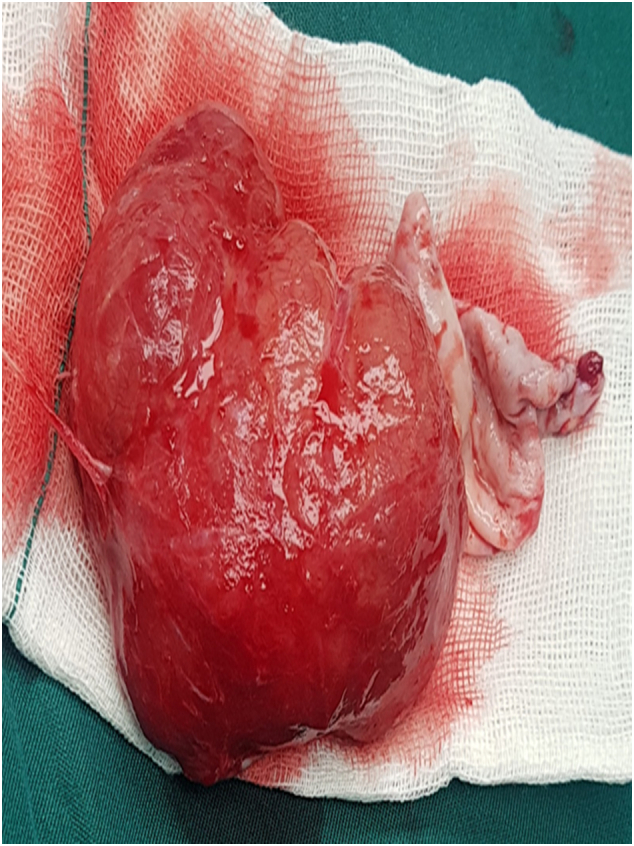
The feature of tumor resected surgically.

**Fig. 4:**
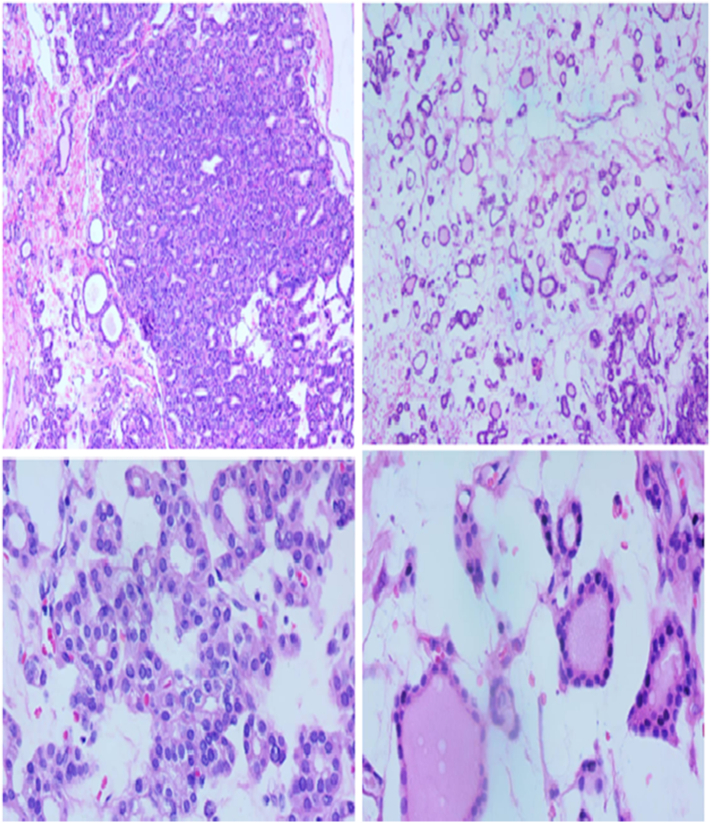
×100 H & E staining feature and ×400 H& E staining feature.

The case described was a 49-year old menopause woman (G4P4L4) referred to our hospital with complaints of abdominal pain, abdominal enlargement and dyspnea. The past medical history, drug history, and family history of the patient were negative. On examination, abdomen was distended without any evidence of vaginal abnormalities. Due to the size of the abdomen, a two-handed examination was not possible. On pulmonary auscultation, there was a decrease in the sound of the lungs. The patient was initially assessed by abdomino-pelvic ultrasonography. In this regard, evidence of abundant ascites was found in the abdomen and pelvis. Baseline laboratory parameters including blood cell count, platelet count, as well as thyroid parameters (TSH, T4) were in the normal range. Heterogeneous solid mass with the diameter of 78 × 58 × 66 mm with cystic regions in the left adnexa, which had severe vascularity in color Doppler examination (ORADS 5) was visualized on ultrasound. Right ovary with the diameter of 22 × 25 mm was seen with normal view. Based on the patient's ultrasound, which concerned for malignancy, further tumor markers and MRI were performed. Pulmonary CT was also requested due to pulmonary symptoms. Assessing tumor markers indicated positivity for CA125 (2599 U/mL) and ROMA Value (14.6) and negativity for HE4, CA19-9, BHCG, CEA, and AFP. In MRI, uterus is normal. There was a 73 × 57 × 89 mm enhancing solid mass lesion in left adnexa. Also, severe ascites and omental infiltration and slightly enhancement were also revealed. The findings were suggestive of malignant mass lesion in left ovary. Right ovary was normal and other pelvic and abdominal organs were unremarkable (Figs. 1 and 2). Moreover, pulmonary CT scanning revealed mild unilateral pleural effusion. To reduce the patient's pain and shortness of breath, ascites fluid was drained. Cytologic exam of the peritoneal fluid was negative for malignancy. The Pap smear test was also negative for intraepithelial lesion or malignancy. Due to the appearance of the mass on imaging, ascites, and elevated tumor markers, the patient underwent laparotomy. The patient's abdomen was explored with a midline incision below the umbilicus. About 4 L of ascites were removed. A mass with adhesion to the pelvic floor was seen on the left (Fig. 3). In the initial examination, no pathological lesion was seen in other parts of the abdomen. The left ovary was sent to the laboratory to be frozen that was ultimately resulted in the definitive diagnosis of struma ovarii in left ovarian (Fig. 4). Pathological assessment of the cervix showed chronic cervicitis. Assessment of omentum also indicated inflamed congested adipose tissue, free from tumor. In immunohistochemistry (IHC), the biomarkers of CK7, TTF1 and PAX8 were found to be positive. Due to the patient's menopause, total abdominal hysterectomy with bilateral salpingo-oophorectomy was performed. Due to severe inflammation of the omentum, a biopsy of the omentum was performed. The patient was discharged after 3 days in good general condition. In following-up, laboratory parameters including thyroid functional indices and CA125 tumor marker were shown to be normalized. In thyroid ultrasonography, only two small thyroid nodules were visible.

After twelve-month follow-up, the patient survived, and no mass was seen on imaging.

## 3 Discussion

Different variants of struma ovarii have been described. In benign strumosis as a rare version of this tumor, mature thyroid tissue may be appeared throughout the peritoneal cavity, but in malignant version named carcinoid, the presence of malignant tissue can be revealed within a struma ovarii [7]. The most common type of malignant form includes papillary thyroid carcinoma occurs in about a third of cases with malignant metastasis in 5 % of them [8]. In epidemiological view, no racial predilection for struma ovarii was found, but this tumor appears commonly in the age range of 40 to 60 years and may be rarely seen before puberty [9]. As the common clinical symptoms, abdominal pain, palpable abdominal mass, abnormal vaginal bleeding, ascites, or pseudo-Meigs syndrome (ascites in the setting of hydrothorax) can be expressed by the patients [10]. Because of nonspecific clinical manifestations, the tumor can be revealed incidentally on pelvic imaging or surgery. Thyroid dysfunction can be also found in 5–8 % of patients [11]. Physical assessment may not be diagnostic, but it may appear with enlarged abdominal palpable mass depending on the location and size of mass. Struma ovarii should be diagnostically differentiated from other gynecological and abdominal more common pathologies such as ectopic pregnancy, hyperthyroidism, and thyrotoxicosis, ovarian cyst, endometrioma, or hydro salpinx [12]. In laboratory study, hematological markers are usually in the normal range. CA125 marker may rise, however, this change is not specifically diagnostics for this tumor [13]. Thyroid function tests are ordered only in patients with symptomatic hyperthyroidism. In imaging evaluation, triple-contrast CT scanning can be helpful to determine the disease extension and involvement of adjacent organs such bowel [14]. However, in CT view, multicystic mass with no or moderate cystic wall enhancement may be revealed. In pathological assessment, struma ovarii has a green-brown and solid appearance, but it may be also found as a cystic feature. It is found commonly unilaterally. In histological assessment, thyroid tissue contains the major component of the tissue as the teratoma [15]. Therapeutically, surgical resection of the ovary is necessary and sufficient to control the benign type of struma ovarii, however in postmenopausal cases with the aim of complete removing the symptoms, total abdominal hysterectomy with bilateral salpingo-oophorectomy can be occasionally indicative [16]. In this regard, following-up for surgical outcomes can be sufficient and commonly leading excellent prognosis. In the present case, the dominant clinical manifestations included abdominal pain along with abdominal enlargement and mild dyspnea without other abdomino-pelvic abnormal findings. The patient was initially suspected to abnormal malignant pathologies in the ovary based on MRI and serum tumor markers assessments, however in further assessment of histological sample along with IHC study led to definitive diagnosis of struma ovarii. The patient was successfully managed surgically without any evidence of recurrence in further assessment.

## Sources of funding

Nothing to declare.

## Ethical approval

Not applicable.

## Consent

Written informed consent was obtained from the patient for publication of this case report and accompanying images. A copy of the written consent is available for review by the Editor-in-Chief of this journal on request.

## Author contribution

1- F.Z.: reporting and interpretation of patient's imaging, Editing the final manuscript
2- A.A.: Main surgeon of the patient
3- F.A.: pathologist, and preparing pathology figures
4- S.GH.: one of the patient surgeon
5- S.N.: collecting data
6- N.Z.: writing and editing the article, corresponding

## Research registration number

N/a.

## Guarantor

Narges Zamani.

## Provenance and peer review

Not commissioned, externally peer-reviewed.

## Declaration of competing interest

Nothing to declare.
